# OncoSNIPE® Study Protocol, a study of molecular profiles associated with development of resistance in solid cancer patients

**DOI:** 10.1186/s12885-021-09134-3

**Published:** 2022-01-06

**Authors:** Sébastien Vachenc, Jessica Gobbo, Sarah El Moujarrebe, Isabelle Desmoulins, Marine Gilabert, Michelle Beau-Faller, Emmanuel Mitry, Nicolas Girard, Aurélie Bertaut, Nelson Dusetti, Juan L. Iovanna, Rahima Yousfi, Fabien Pierrat, Roman Bruno, Adèle Cueff, Romain Boidot, Philippe Genne

**Affiliations:** 1 Oncodesign SA, 18-20 rue Jean Mazen, 21079 Dijon Cedex, France; 2 Centre Georges-François Leclerc, 1 rue Professeur Marion, 21079 Dijon Cedex, France; 3 Inserm 1231, 8 Boulevard Jeanne d’ARC, 21000 Dijon, France; 4 Institut Paoli-Calmettes, 232 Bd de Sainte Marguerite, 13009 Marseille, France; 5 Hôpitaux Universitaires de Strasbourg, Avenue Molière, 67000 Strasbourg, France; 6 Institut Curie, 26 Rue d’Ulm, 75005 Paris, France; 7 Cancer Research Center of Marseille, CRCM, Inserm, CNRS, Paoli-Calmettes Institut, Aix-Marseille University, Marseille, France; 8 Acobiom SAS, 1682 rue de la Valsière Cap Delta Biopôle Euromédecine II, 34790 Grables, France

**Keywords:** Early/ late resistance marker, NGS, Immunological profile, Triple negative breast or Luminal Breast Cancer, Non small-cell lung cancer, Pancreatic ductal adenocarcinoma

## Abstract

**Background:**

Nowadays, evaluation of the efficacy and the duration of treatment, in context of monitoring patients with solid tumors, is based on the RECIST methodology. With these criteria, resistance and/or insensitivity are defined as tumor non-response which does not allow a good understanding of the diversity of the underlying mechanisms. The main objective of the OncoSNIPE® collaborative clinical research program is to identify early and late markers of resistance to treatment.

**Methods:**

Multicentric, interventional study with the primary objective to identify early and / or late markers of resistance to treatment, in 600 adult patients with locally advanced or metastatic triple negative or Luminal B breast cancer, non-small-cell lung cancer or pancreatic ductal adenocarcinoma. Patients targeted in this study have all rapid progression of their pathology, making it possible to obtain models for evaluating markers of early and / or late responses over the 2-year period of follow-up, and thus provide the information necessary to understand resistance mechanisms. To explore the phenomena of resistance, during therapeutic response and / or progression of the pathology, we will use a multidisciplinary approach including high-throughput sequencing (Exome-seq and RNAseq), clinical data, medical images and immunological profile by ELISA. Patients will have long-term follow-up with different biological samples, at baseline (blood and biopsy) and at each tumoral evaluation or tumoral progression evaluated by medical imaging. Clinical data will be collected through a dedicated Case Report Form (CRF) and enriched by semantic extraction based on the French ConSoRe (Continuum Soins Recherche) initiative, a dedicated Semantic Clinical Data Warehouse (SCDW) to cancer.

The study is sponsored by Oncodesign (Dijon, France) and is currently ongoing.

**Discussion:**

The great diversity of intrinsic or acquired molecular mechanisms involved in resistance to treatment constitutes a real therapeutic issue. Improving understanding of mechanisms of resistance of cancer cells to anti-tumor treatments is therefore a major challenge. The OncoSNIPE cohort will lead to a better understanding of the mechanisms of resistance and will allow to explore new mechanisms of actions and to discover new therapeutic targets or strategies making it possible to circumvent the escape in different types of cancer.

**Trial registration:**

Clinicaltrial.gov. Registered 16 September 2020, https://clinicaltrials.gov/ct2/show/NCT04548960?term=oncosnipe&draw=2&rank=1 and ANSM ID RCB 2017-A02018-45.

## Background

Precision medicine is considered to be one of the major issues in patient care. A lot of researches have already proven successful implementation of targeted therapies including immunotherapies that offers to patient’s improved response and survival rates. But despite these major therapeutic advances, resistance to anti-cancer treatment is a major obstacle in the care of patients (1,2). Indeed, to date, many patients still die of cancer with 9.6 million deaths worldwide in 2018. Nowadays, improving understanding of mechanisms of resistance of cancer cells to anti-tumor treatments is therefore a major issue. The great diversity of molecular mechanisms involved in the phenomena of resistance to treatment, whether intrinsic (de novo, or primary) or acquired (secondary), constitutes a real therapeutic challenge (3, 4, 5). Indeed, a better understanding of the mechanisms of resistance would make it possible to explore new therapeutic strategies making it possible to circumvent these phenomena of escape in different types of cancer. The OncoSNIPE project was developed in this context. The objective of this project is to identify early and / or late markers of resistance to treatment in 3 different pathologies affected with resistance issues: triple negative breast cancer or Luminal B, locally advanced or metastatic non-small-cell lung cancer and pancreatic ductal adenocarcinoma (PDAC). In this project, in order to best cover the diversity of mechanisms involved in these resistances, we propose a multidisciplinary approach with clinical, genomic, transcriptomic and immunological dimensions.

## Methods/ Design

### Study population

Adult patients with a diagnosed triple negative or Luminal B breast cancer, locally advanced or metastatic non-small-cell lung cancer or PDAC and managed at Georges-François Leclerc Cancer Center (Dijon), Curie Institute (Paris), Paoli Calmettes Institute (Marseille), Haute-Pierre Hospital (Strasbourg), Godinot Institute (Reims), Léon Bérard Center (Lyon), Lorraine Cancer Institute (Nancy), APHP-Beaujon Hospital (Clichy), F-Mitterand Dijon-Bourgogne University Hospital Center (Dijon), La Miletrie University Hospital Center (Poitiers) and Besancon University Hospital Center (Besançon).

All inclusion and exclusion criteria are described in Table 1.

**Table 1:** OncoSNIPE study inclusion and exclusion criteria

**Inclusion criteria**	
General inclusion critreria	Adult patient, 18 years of age or older
	Naive chemo patient
	Performans status: 0,1 or 2
	Life expectancy > 3 months
	Subject affiliated to a social security and health insurance scheme
	Subject having dated and signed informed consent
	For women of childbearing age (negative pregnancy test): effective contraception
Specific inclusion criteria for Pancreatic ductal adenocarcinoma	Patient with histologically proven PDAC or Patient receiving a biopsy, as part of the usual care of the patient: Either from the primary tumor, Either a metastasis for a strong suspicion of locally advanced or metastatic PDAC
	With advanced or metastatic tumors (liver, lungs, peritoneum, others) that cannot benefit from local or locoregional treatment;
	Presence of target lesion (s) measurable according to RECIST criteria
	Patient who cannot be treated by surgery or radiotherapy
	Rate of tumor cells observed on FFPE biopsies must be ≥ 30%, otherwise, microdissection enrichment must be carried out to reach this threshold
Specific inclusion criteria for Lung cancer	Patient with histologically proven non-small cell lung cancer
	Locally advanced stage IIIB or IV
	Treatment with chemotherapy, targeted therapy, immunotherapy
	Tissue available after analysis of the usual biomarkers in the event of a non-epidermoid tumor
	Rate of tumor cells observed on FPPE biopsies must be ≥ 30%
	Presence of measurable target lesion or disease assessable according to RECIST criteria
Specific inclusion criteria for Breast cancer	Breast cancer of recent diagnosis, histologically proven
	Triple negative breast cancer: negativity of estrogen and progesterone receptors in the tumor (< 10%), absence of HER2 overexpression according to the IHC classification (score 0 or 1 +) and / or FISH negative or LumB: RO positive, RP positive or negative, HER2 negative, high proliferation;
	Stage I to III for triple negative breast cancer (including stage T4d = inflammatory cancer), Stage II or III of the UICC classification for LumB
	Non-metastatic patient (M0 according to TNM classification)
	Rate of tumor cells observed on FFPE biopsies must be ≥ 30%
	Patient who cannot be treated exclusively by surgery or radiotherapy
**Exclusion Criteria**	
General Exclusion criteria	History of chemotherapy (except adjuvant completed for more than at least 6 months) or radiotherapy
	Patient whose monitoring and treatment will not be carried out in the study health establishments;
	Tumor not histologically proven;
	Life expectancy of less than 3 months
	Pregnancy or breastfeeding
	Refusal to participate in the trial
	Persons deprived of their liberty, persons under guardianship or curatorship
	Inability to submit to the medical follow-up of the test for social or psychological reasons
	No affiliation to a social security scheme or state medical aid (AME) or universal medical coverage (CMU)
	Any condition for which participation in the protocol would present a risk or which would not make it possible to comply with the requirements of the protocol according to the investigator
	History of other cancers in the last 5 years except cervical cancer and skin cancer of the basal or epidermoid cells treated
	Known HIV seropositivity
Specific exclusion criteria for Pancreatic ductal adenocarcinoma	Other histologies: neuroendocrine cancer, acinary cell carcinoma, pancreatic metastasis of another cancer
	Patient who cannot benefit from chemotherapy (Performans status (PS) 3—4);
	Other progressive cancer during the management of PDAC;
	Co-morbidities: Significant and / or uncontrolled pathologies or other conditions that may affect participation in the study, such as: unstable angina, symptomatic or uncontrolled arrhythmia requiring treatment, uncontrolled hypertension, congestive heart disease class NYHA II, III or IV, myocardial infarction or stroke in the 6 months before the study; Active or uncontrolled infection or pathology (compromised) compromising the ability to assess the patient or allow the patient to complete the study; Benign illnesses not controlled or whose control could be compromised by the treatment under study, such as severe diabetes, not controlled by medical treatment
Specific exclusion criteria for Breast cancer	Non-operable or metastatic breast cancer
	History of breast cancer treated
	Co-morbidities: Significant and / or uncontrolled pathologies or other conditions that may affect participation in the study, such as: unstable angina, symptomatic or uncontrolled arrhythmia requiring treatment, uncontrolled hypertension, congestive heart disease class NYHA II, III or IV, myocardial infarction or stroke in the 6 months before the study; Active or uncontrolled infection or pathology (compromised) compromising the ability to assess the patient or allow the patient to complete the study; Benign illnesses not controlled or whose control could be compromised by the treatment under study, such as severe diabetes, not controlled by medical treatment
Specific exclusion criteria for Lung cancer	Small cell lung cancer
	Stage I to IIIa non-small cell lung cancer
	Treatment by curative radiotherapy or radio-chemotherapy

### Study objectives and endpoints

The main objective of this study is to identify biomarkers of resistance to treatment in patients with triple negative or Luminal B breast cancer, locally advanced or metastatic non-small cell lung cancer or PDAC.

Analyses will be performed in each cohort independently. The primary endpoint will be a combinatory analysis of genomic, transcriptomic, clinical, medical images and immunological profile realized by ELISA and NGS (Next Generation Sequencing; Exome-seq, RNA-seq). Markers can be early or late biomarkers. The early markers will be markers present at baseline, the late markers will be the ones appearing during the follow-up. Resistance is defined as a tumoral nonresponse according to the RECIST (Response Evaluation Criteria In Solid Tumor) criteria or absence of Pathologic Complete Response (pCR).

The secondary objectives are to (i) identify therapeutic targets; (ii) determine the prognostic impact of the resistance markers on progression-free survival (PFS) and overall survival (OS),with PFS defined as the time between inclusion and progression (according to RECIST criteria or pCR) or death whatever the cause, and OS defined by the time between inclusion or death whatever the cause; (iii) identify the clinical, biological and/or genomic characteristics of long survivors (ie Patients who are still responding to the first line of treatment at the end of the 2-year follow-up.

### Study design

**Fig. 1:**
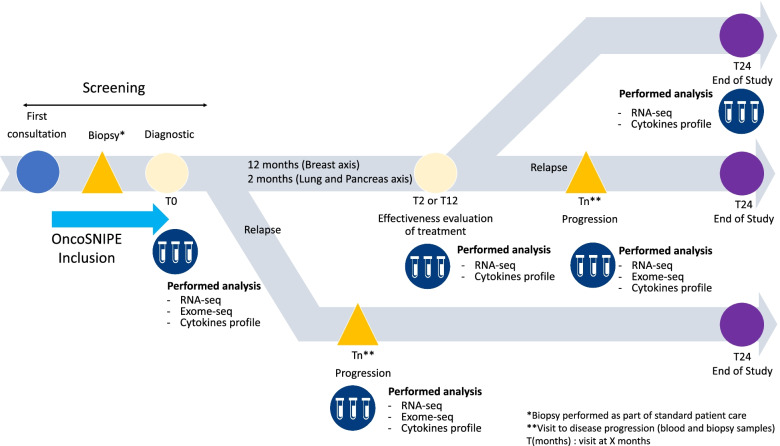
Study Design

Prospective, multi-centric, non-randomized study. Study inclusion period will be four years, each patient will be followed for 2 years and study total duration will be 6 years. Study design is depicted in Fig. 1.

Investigators will receive an informative notice on OncoSNIPE study and will provide eligible patients with laboratory procedures.

1. *Sampling*
  - Initial sampling
    Prior to any treatment tumor biopsy and 10 ml of blood (2*2.5 mL RNA PAXgene tubes, 5 mL dry tubes) will be collected. Tumors biopsy will be collected in standard care for histologically proven breast, lung and PDAC patients with formalin-fixed paraffin-embedded (FFPE) standard procedure and PDAC-derived organoid will be produced for non-histologically proven pancreas cancer patients before the clinical study inclusion. RNA-seq and Exome-seq will be performed on tumor biopsy. Cytokine profile and RNA-seq will be performed on blood samples.
  - Follow-up
    Sample collection schedule will depend on treatment standard of care: 10 ml blood sample (2*2.5 ml RNA PAXgene tubes, 5 ml dry tubes) analysis will be collected at 2 months (T2) for pancreatic and lung cancer and 12 months (T12) for breast cancer. Cytokine profile and RNA-seq will be performed.
    Sample schedule will be maintained even if toxicity leads to treatment changes.
  - Disease progression
    If disease progression is observed, blood and tumor biopsy will be collected: 10 ml blood (2*2.5 ml RNA PAXgene tubes, 5 ml dry tubes). Tumor biopsies will be collected in standard care for breast and lung cancer. RNA-seq and Exome-seq will be performed on tumor biopsies. Cytokine profile and RNA-seq will be performed on blood samples.
2. Sample processing
  PAXgene tubes will be kept at RT (15–25 °C) at least 2 h and then placed at -20 °C 24 h to 72 h. Tubes will then be transferred at -80 °C until use.
  Blood samples collected in dry tubes will be kept for a maximum of 1 h at RT (15–25 °C). After centrifugation (1200 g, 15 min, RT) serum will be aliquoted (about 10 tubes of 250 µL) and will be store at -80 °C.
  PDAC-derived organoids were obtained from patients with unresecable tumors by Endoscopic Ultrasound Fine Needle Aspiration (EUS-FNA). Biopsies will be digested with Tumor Dissociation Kit (Miltenyi Biotec) at 37 °C for 5 min, then incubated with Red Blood Cell Lysis Buffer (Roche) and washed 2 times with PBS. The digested samples will be transferred into a tissue strainer 100 μm and will be placed into 12-well plate coated with 150 μl GFR matrigel (Corning). The samples will be cultured with Pancreatic Organoid Feeding Media (POFM) consisting of Advanced DMEM/F12 supplemented with 10 mM HEPES (Thermo-Fisher); 1 × Glutamax (Thermo-Fisher); penicillin/streptomycin (Thermo-Fisher); 100 ng/ml Animal-Free Recombinant Human FGF10 (Peprotech); 50 ng/ml Animal-Free Recombinant Human EGF (Peprotech); 100 ng/ml Recombinant Human Noggin (Biotechne); Wnt3a-conditioned medium (30% v/v); RSPO1-conditioned medium (10% v/v); 10 nM human Gastrin 1 (Sigma Aldrich) 10 mM Nicotinamide (Sigma Aldrich); 1.25 mM N acetylcysteine (Sigma Aldrich); 1 × B27 (Invitrogen); 500 nM A83-01 (Tocris); 10.5 μM Y27632 (Tocris). The plates will be incubated at 37 °C in a 5% CO_2_ incubator, and the media will be changed every 3 or 4 days.
3. Sample analysis
  - Tumors analysis:
    FFPE tumors for breast and lung cancer will be analyzed by a pathologist to determine the percentage of tumor cell content. Only samples with more than 30% of cancerous cells and successful PDAC-derived organoids will be analyzed.
    DNA will be extracted using the Maxwell RSC DNA FFPE kit (Promega) according to the manufacturer’s protocol. DNA will be quantified using fluorometric assay with a Qubit device. RNA will be extracted using the Maxwell RSC RNA FFPE kit (Promega) according to the manufacturer’s protocol. RNA quality and quantity will be assessed by spectrophotometry with absorbance at 230, 260 and 280 nm.
    i. Exome analysis on tumor samples
      Two hundred nanograms of genomic DNA will be fragmented with a Covaris device to obtain fragments around 300 bp. Then, libraries will be constructed and captured by using SureSelect Human All Exon v6 kit (Agilent), following the manufacturer’s protocol. Paired-end (2 × 111 bases) sequencing will be performed on a NextSeq500 device (Illumina) as described previously (6–10) with a mean target coverage of 100X.
    ii. RNA sequencing on tumor samples
      Ribosomal RNA depleted RNA will be used for library preparation with the NEBNext Ultra II Directional RNA library prep kit for Illumina according to the manufacturer’s instructions (New England Biolabs). RNA sequencing will be performed on a NextSeq500 device (Illumina) as described previously (6;10–13). The libraries will be sequenced with paired-end 76–base pair ‘reads’ with a target of 30 million of reads per sample.
      b) Blood samples analysis:
    iii. RNA sequencing on blood samples
      RNA from PAXgene blood samples will be extracted using the Maxwell RSC simply RNA blood kit (Promega) according to the manufacturer’s protocol. RNA quality and quantity will be assessed by spectrophotometry with absorbance at 230, 260 and 280 nm.
      Ribosomal and globin RNA depleted RNA will be used for library preparation with the NEBNext Ultra II Directional RNA library prep kit for Illumina according to the manufacturer’s instructions (New England Biolabs). RNA sequencing will be performed on a NextSeq500 device (Illumina). The libraries will be sequenced with paired-end 76–base pair ‘reads’ with a target of 30 million of reads per sample.
    iv. Cytokine profiling on blood samples
      Cytokines will be analyzed using the multiplex assay provided by Meso Scale Discovery MSD®. The principle of the multiplex assay resides on specific binding between unique linkers organized onto unique spot, which couples with specific biotinylated captures antibodies (developed by MSD®). Cytokines in the sample bind to the specific biotinylated capture antibodies. Then, detection antibodies conjugated with electroluminescent labels bind to the analyte to complete sandwich immunoassay. Once this step done, the QUICKPLEX SQ 120 (MSD®) applies a voltage to the microplate electrodes and the captured labels emit light. The instrument measures the emitted light from each spot, which is proportional to the analyte present in the sample and provide a quantitative measure.
4. Bioinformatics analysis
  - NGS (RNAseq and Exome libraries) quality control and preprocessing of FASTQ files are controlled to provide clean data for downstream NGS analysis (number of reads, length of the insert, duplication rate, percent of GC, coverage…). Before analysis, the library files are cleaned up by removing bad quality bases, unknown bases “N” and too short sequences, and by trimming artificial sequences such as adapters.
  - **RNAseq analysis:** Sequenced data is mapped on the human genome, using the "hg19" version of the human genome and version 92 of the Ensembl database for annotation features. The mapping is performed by the STAR software with paired-end parameters. Through a double approach, we count genes and exons separately. The Htseq-count program is run two times, once for measuring the number of genes and the other for measuring the exons. The counting of both Exon and Gene data is intended to increase the mapping quality and the accuracy of the analysis. We perform a differential expression analysis (DE) considering potential genes/exons with the software R version 4.0.3 with a double-crossed analysis using both edgeR v3.32.0 and DESeq v1.30.0 packages. Results from both methods will be crossed to assure a better reliability.
  - **Exome analysis:** Sequenced data will be mapped on the human genome, using the "hg19" version of the human genome and version 92 of the Ensembl database for annotation features. The mapping will be performed by the BWA software with paired-end parameters and the 'MEM' option due to the large size of the human genome and the need to allocate a large amount of RAM memory for the execution of the software. After mapping reads to the reference genome, the workflow will removed duplicates before variant calling to mitigate biases introduced by data generation steps such as PCR amplification. After that, base quality scores will be recalibrated, because the variant calling algorithms rely heavily on the quality scores assigned to the individual base calls in each sequence read. For variant calling, different softwares will be used to detect both SNVs (Single Nucleotide Variant) and InDels (Sequence Insertion or Deletion): Mutect2 and GATK HaplotypeCaller for SNVs detection, and GATK HaplotypeCaller and Pindel for InDels detection.

### Data collection

The following data will be collected during the study, at the time of inclusion and during medical follow-up: demographic and clinical data (age, sex, weight, height, performance status, SBR, …), medical history, date of diagnosis, 1^st^ and 2^nd^ lines of treatments, disease evaluation reports (biological, imaging,…), will be sent as a standard report from the laboratory in charge of these analysis. All data will be recorded in the CRF (CSOnline 7.0.204.3 ClinSight®).

### Sample size

- PDAC axis:

The expected response rate at 2 months is 30%.The probability modelized is non response to treatment with the assumption of an area under the ROC (Receiver Operating Characteristic) curve (AUC, Area Under de Curve) between 80 and 85%, a number of 200 subjects, i.e. 60 responders and 140 resistants (non-responders) will allow us to reach a power between 82 and 100% to prove a difference of 0.10 to 0.15 points against a theoretical AUC of 0.70, with a unilateral alpha risk of 5%.

To get 200 assessable patients for PDAC axis, a total of 400 patients should be included to anticipate a rate of non-evaluable patients of 50%.

Patients not assessable for the pancreas axis will be replaced and are defined as follows: premature exit (death) from study before treatment, patient who received less than 2 treatment cycles, patient who left studies prematurely before carrying out the efficacy visit (2 months after the start of treatment) or progression and patient with unsuccessful PDAC-derived organoid development.

b) Lung cancer axis:

The expected response rate at 2 months is 30%. The expected response rate at 2 months is 30%. The probability modelized is non response to treatment with the assumption of an area under the ROC curve (AUC) between 80 and 85%, a number of 200 subjects, i.e. 60 responders and 140 resistants (non-responders) will allow us to reach a power of between 82 and 100% to prove a difference of 0.10 to 0.15 points against a theoretical AUC of 0.70, with a unilateral alpha risk of 5%.

To get 200 assessable patients for lung cancer axis, a total of 260 patients should be included to anticipate a rate of non-evaluable patients of 30%.

Patients not assessable for the lung axis will be replaced and defined as follows: prematurely exit (death) from study before treatment, patient having received less than 2 treatment cycles, patient who left studies prematurely before carrying out the efficacy visit (3 months after the start of treatment) or progression (excluding patients always responding after 24 months of follow-up).

iii) Breast cancer axis:

The expected response rate at 2 years is 50%. The probability modelized is non response to treatment with the assumption of an area under the ROC curve (AUC) between 80 and 85%, a number of 200 subjects, i.e. 100 responders and 100 resistants (non-responders) will allow us to reach a power of between 86 and 100% to prove a difference of 0.10 to 0.15 points against a theoretical AUC of 0.70, with a unilateral alpha risk of 5%.

To get 200 assessable patients for breast cancer axis, a total of 230 patients should be included to anticipate a rate of non-evaluable patients of 15%.

Patients not assessable for the breast axis will be replaced and defined as follows: prematurely exit (death) from study before treatment, patient having received less than 2 treatment cycles, patient who left studies prematurely before carrying out the efficacy visit (3 months after the start of treatment) or progression (excluding patients always responding after 24 months of follow-up).

Non-assessable patients will be replaced.

### Statistical analysis

The analyses will be carried out independently for each cohort. A full statistical plan will be written before database lock. The analysis will be performed under SAS 9.4.

## Discussion

Despite major therapeutic advances driven by precision medicine initiatives in the last two decades, resistance to cancer therapy remains a major obstacle in the patient care.

The great diversity of molecular mechanisms involved in the phenomena of resistance to treatment, whether intrinsic (de novo, or primary) or acquired (secondary), constitutes a real therapeutic issue. Understood the diversity of molecular mechanisms which sustain the resistance of cancer cells to anti-tumor treatments is therefore a major challenge and the main objective of the OncoSNIPE® collaborative research program.

The deep characterization of the OncoSNIPE® cohort and the semantic enrichment based on the implementation of bio-informatics, artificial intelligence, statistical learning and semantic enrichment approaches, will lead to a better understanding of the mechanisms of resistance and will allow to explore new mechanisms of actions and to discover new therapeutic targets or strategies making it possible to circumvent these phenomena of escape in different types of cancer.

## Data Availability

This section is not applicable.

## Abbreviations

AUC: Area Under de Curve
ConSoRe: “Continuum Soins Recherche”
CRF: Case Report Form
DE: Differential expression analysis
EUS-FNA: Endoscopic Ultrasound Fine Needle Aspiration
FFPE: Formalin-fixed paraffin-embedded
NGS: Next Generation Sequencing
RECIST: Response Evaluation Criteria In Solid Tumors
OS: Overall survival
PDAC: Pancreatic ductal adenocarcinoma
pCR: Pathologic Complete Response
PFS: Progression-free survival
RNA: Ribonucleic acid
ROC: Receiver Operating Characteristic
SCDW: Semantic Clinical Data Warehouse
